# Effect of Sugar Substitution with Steviol Glycosides on Sensory Quality and Physicochemical Composition of Low-Sugar Apple Preserves

**DOI:** 10.3390/foods9030293

**Published:** 2020-03-05

**Authors:** Marlena Pielak, Ewa Czarniecka-Skubina, Artur Głuchowski

**Affiliations:** Department of Food Gastronomy and Food Hygiene, Institute of Human Nutrition Sciences; Warsaw University of Life Sciences (WULS-SGGW), 159C Nowoursynowska St., 02-776 Warsaw, Poland; marlena_pielak@sggw.pl (M.P.); artur_gluchowski@sggw.pl (A.G.)

**Keywords:** steviol glycosides (SGs), apple preserves, low-sugar products, sensory quality, physicochemical composition

## Abstract

The purpose of this study was to determine the sensory profile and consumer response, as well as physicochemical properties of low-sugar apple preserves (with or without gelling agent or acidity regulator), in which sugar was replaced with varying amounts of steviol glycosides (SGs). According to the analytical assessment and consumer tests’ results, the reduction of sugar by SGs use in the apple preserves without food additives was possible at a substitution level of 10% (0–0.05 g/100 g). Consumers’ degree of liking for sugar substitution with SGs was high, up to 40% (0.20 g/100 g) in the preserves, with the use of pectin and citric acid. Higher levels of sugar substitution with the SGs resulted in flavor and odor deterioration, such as a metallic flavor and odor, a bitter taste, an astringent oral sensation, and a sharp odor. The use of food additives (pectin, citric acid) in apple preserves, allowed the SGs substitution level to be increased. The preserves (Experiment I, II, III) with higher sensory ratings were subjected to physicochemical tests. Physical and chemical analysis of low-sugar products with sucrose substitution by SGs at the level of 10%, 30%, 40% showed their good technological quality. The results demonstrated the possibility of substituting sugar with steviol glycosides to produce energy-reduced apple preserves, with acceptable sensory quality and good physicochemical properties.

## 1. Introduction

The consumption of sweeteners has steadily increased over the last decades. A large proportion of sugar is consumed from processed food; therefore, the World Health Organization [1] recommends limiting the consumption of free sugar in the diet to less than 10% of total energy needs and to less than 5% for additional health benefits. Due to the health implications of consuming a diet high in sugar, such as obesity, diabetes, and heart disease, health professionals have put pressure on food manufacturers to reduce the amount of sugar in processed food. The food industry has reduced the sugar content in the production of food or ingredients that mimic the functional property of sugar (artificial or natural ingredients that have lower or zero calories). 

In recent years, the demand for natural sweeteners with low calorific value, an acceptable taste, and healthy qualities which could replace saccharose was increased [2]. To limit sugar, the alternative sweeteners: semisynthetic (e.g., lactitol, maltitol, xylitol), synthetic (e.g., aspartame, acesulfame K, saccharinate, cyclamate), as well as natural substances (e.g., thaumatin, curculin, steviol glycosides) were used by producers. Consumers are also increasingly interested in reducing the sugar content of products. According to other authors [3], consumers declared taking up the activities towards reducing sugar intake in their diet mostly due to health-related reasons. It was emphasized in particular by women. The most frequent way to limit the amount of sugar in the diet consisted of choosing sweeteners, mainly stevia and xylitol.

Determining the precise clinical benefits of reducing added sugars requires long-term research. For this reason, the search for novel sweeteners, especially natural sweeteners, is ongoing [4]. Commercial sweeteners are usually made of a combination of natural and artificial sweet substances. It is advisory to eliminate artificial sweeteners from the diet of some people, especially those pregnant and during lactation [5]. 

For the time, relatively few sweet-tasting plant-derived natural products have been launched commercially as sucrose substitutes [6]. The major topic addressed in the publications is the public or personal pressure on the consumer to make the switch to more natural low- or zero-calorie, alternatives of sugar. The two botanical sweeteners that have enjoyed popularity in just a few years are stevia (*S. rebaudiana*) and luo han guo (*S. grosvenorii*) [7].

Steviol glycosides sourced from the *Stevia rebaudiana* plant in 2011 were approved by the European Food Safety Authority for use in food and maintain the interest of technologists [8,9]. As consumer preferences continued to shift toward natural products, the consumption of steviol glycosides is expected to increase more than other low-calorie sweeteners [10]. 

Natural alternatives to ‘white sugar’ and artificial sweeteners should come from a natural origin, mimic the taste of sugar, and possess appropriate technological properties like the sugar substitute Steviol glycosides (SGs). They are derived from the *Stevia rebaudiana Bertoni* plant and mainly consist of stevioside (around 65%) and rebaudioside A (around 25%). Others: rebaudioside B, C, D, E, F, dulcoside A, C, and steviolbioside are less important [11]. In a very pure form, they are characterized by a high intensity of sweetness and are non-calorific [12]. The most promising flavor profiles have formulas with highly purified rebaudioside A (99%) [13]. The rebaudioside A exhibits a high sweetening potency, as well as one of the most preferred flavor profiles, giving the least bitter and sour flavor [14,15], while stevioside is 200-450 times sweeter than sucrose, responsible for the bitter and licorice aftertaste, and enhances the odor. The bitter taste of stevioside is less noticeable when mixed with rebaudioside A in equal proportions [16,17,18]. 

Since 2011, steviol glycosides can be used as a food additive in the EU. The established maximum amount permitted in preserves is 200 mg/kg level expressed as a steviol equivalent [10]. Steviol glycosides’ technological properties as a sugar substitute for preserves are good because of its high stability at a broad pH (2–9) level and during storage in different conditions, light resistance, and thermostability at temperatures up to 200 °C. Moreover, during long-term storage, the sweetness of the product does not diminish [19,20,21]. 

SGs are used by food producers in snacks, pickled vegetables, dried seafood, soy sauces, miso production [22], in low calorie beverages [23], candy, chewing gum, ice cream, tea, yoghurt [24,25], baked goods [26,27,28], muffins [27,28,29,30,31], dried fruits [32,33]. Few publications focused on fruit purée and jams [34,35,36,37,38,39]. 

Substituting sugar with stevia in apple preserves seems to be a good option because apples are very popular and grown widely throughout Europe [40] as well as the fact that they have a positive effect on reducing the risk of developing type 2 diabetes and aid in weight loss [28]. Apple preserves in many countries are used in the form of mousse, jams, compotes, marmalade, purees for cakes, such as apple pie. Due to the low cost of apples, they are used as the main ingredient in mixed fruits jams. Moreover, in the era of industrial food production, consumers are increasingly looking for natural “clean label” products, produced in a traditional way, without or with limited amounts of food additives. For example, there were no preserves, especially apple preserves, with glycosides on the Polish market [41] and we did not find a publication about apple preserves with replacement sugar by SGs. These findings may be used in the future for other fruits preserves. 

The aim of the study was to determine the effects of sugar substitution with steviol glycosides on the sensory profile and consumer response toward apple preserves with or without additives (pectin, citric acid) as well as to evaluate the physicochemical properties of selected preserves. A hypothesis was made that a high level of sugar substitution with steviol glycosides deteriorated the sensory quality of the products to an unacceptable level by consumers, and their taste and flavor improvement by use of food additives (pectin, citric acid) is possible.

## 2. Materials and Methods

### 2.1. Materials

**Figure 1:**
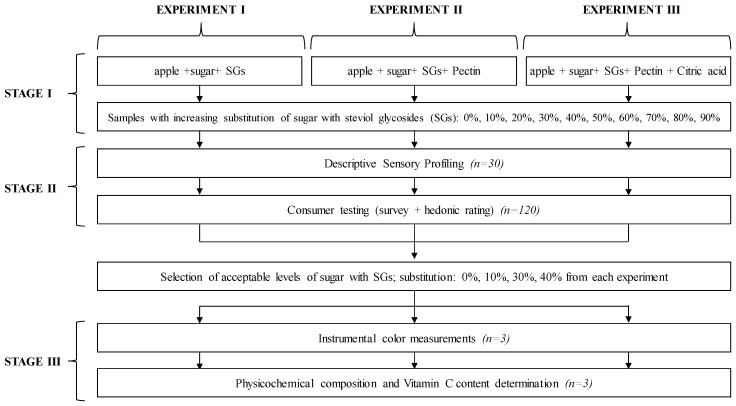
Study design.

*Experimental design*. The study consisted of three experiments that had the same three-stage design (Figure 1). In Experiment 1, only those apple preserves with an addition of a mixture of sugar and steviol glycosides (SGs) were prepared and evaluated. In Experiment 2, amidated pectin was also added to the preserves, while in Experiment 3, aside from the earlier mentioned ingredients, an aqueous solution of citric acid was used. At the first stage, the composition of apple preserves was developed and the preserves were prepared. In the second stage, sensory profile and consumer acceptance were assessed. In the third stage, instrumental color, as well as physicochemical composition and Vitamin C content in selected preserves was measured. 

#### 2.1.1. Preparation of Apple Preserves

Gloster apples (*Malus domestica ‘Gloster’)*, which were used to produce the preserves, were derived from the harvests of the University orchards and were prepared using a homemade method, in accordance with GMP (Good Manufacturing Practice) guidelines. The comminuted apple flesh was mixed with other ingredients, boiled, packed in sterile jars, and pasteurized for 10 min when the temperature reached 85 °C. Low-sugar samples (L samples) had an apple pulp/sugar ratio of 10:5 (m/m) which was accepted by the sensory panel in preliminary studies. Then, 1 g of sugar was substituted with 0.01 g of steviol glycosides at 0, 10, 20, 30, 40, 50, 60, 70, 80, 90 percent levels, based on the ratio established in the initial studies. 

The composition of all the versions of the preserves is listed in Table 1. For sweetening, a white crystal sugar (Diamant, Pfeifer & Langen Poland S.A, Gostyń, Poland) was used. For sugar substitution, high purity (95.1%, HPLC) powdered steviol glycosides (Stevia Natura SAS firm, Riom, Auvergne, France) of steviol equivalent—363 (mg/g) was used. 

A powdered amidated citrus-apple pectin (Agnex firm, Bialystok, Poland) with a 30%–35% degree of esterification (DE) and 15%–20% of amidation (DA) was used. Low-sugar jams with an extract below 60% and artificially sweetened low-energy products and preserves with SGs require the use of low-methylated (LM) pectin, mainly amidated. LM is often used in low-sugar products to obtain gel-forming properties with or without only a small amount of sugars in the presence of Ca^2+^ and at a pH level <3.5 [42]. The pectin formula solution was prepared by mixing a 3 g pectin (per 1 kg of apples) with sugar and water. The amount of citric acid powder (Agnex firm, Białystok, Poland) based on the acidity balance was calculated. The total extract of low-sugar preserves was 45.2%–47.5%. 

#### 2.1.2. Sample Presentation for Sensory Evaluation

Apple preserves (30g) were placed in plastic containers, then covered with a lid, and put in a Styrofoam box with three pockets, coded and delivered for assessment at a temperature of 21 °C ± 1 °C. 

### 2.2. Sensory Analytical Method

Descriptive Sensory Profiling—using a quantitative descriptive analysis was evaluated by a 10-person assessing panel. Panelists held expert qualifications according to PN-EN ISO 8586:2014-03 [43] and had extensive experience in sensory analytical assessments. The conditions for the assessment were determined in accordance with Civille et al. [44] and performed in a sensory laboratory that meets the requirements of PN-ISO 8589:2007 [45]. The selection of quality descriptors for analysis was carried out in accordance with the procedure of PN-ISO 13299:2016 [46]. Nineteen attributes were assessed on an 100 mm unstructured graphical scale (Table 2). A verbal definition list with standardized vocabulary was developed and provided to assessors during evaluation. 

Due to the diversity of assessed products in three experiments, the consistency of the product was deliberately abandoned so that it would not affect the panelists’ assessments. The experts were informed not to consider the consistency in assessing the degree of sensory balance of the product. The evaluation was performed in 30 sessions and during each of them, three samples in random order were assessed. 

### 2.3. Consumer Testing

Consumer tests consisted of questionnaires aimed to determine the factors affecting consumer responses and a sensory rating. Respondents were asked to indicate the characteristics of the product (color, flavor, odor, consistency, other) that influenced their assessment. They were also asked to estimate the frequency of fruit preserve consumption (2–3 times a week, occasionally, or not at all), the reasons for limiting sugar consumption (health reasons, to keep fit, sensory aspects, others), as well as gender and age. To quantify the overall degree of consumer liking, the 9-point classical hedonic scale: from 9 = like extremely to 1 = dislike extremely was used [47].

Volunteers who agreed to participate in a product test were invited to the sensory laboratory. Participants started testing after completing the survey, thereafter they received an evaluation form with written procedure and samples of preserves. Due to a large number of samples, they were evaluated by consumers in 6 sessions, during which 5 samples in random order were assessed. During the experiment, testing was conducted in a group of 120 untrained people between the ages of 18–45. The group of surveyed consumers was dominated by women (77.1%) and people younger than 25 (83.7%).

The respondents were free to participate in consumer tests. Based on the fact that the research is non-invasive and the details of the participants remain undisclosed, this research does not fall within the remit of the Helsinki Declaration.

### 2.4. Instrumental Color Measurements

Instrumental color measurements were made using a spectrophotometer (CM-2300d, Konica-Minolta GmbH, Langenhagen, Germany). The equipment was set up for standard illuminate D65, (10° observer angle) and calibrated using a standard white reflector plate (Minolta Technical Note 1994). The measurement was performed in three replications, both in raw apple pulp and in ready apple preserve. Results were expressed using a CIE (L*a*b*) system. The following parameters were set: L*—lightness (L* = 0 black, L* = 100 white), a*—the proportion of green (a* < 0) or red (a*>0), b*—the proportion of blue (b* < 0) or yellow (b* > 0). The differences (Δ) between given coordinates were calculated by subtracting the colorimetric values of ready preserve sample from the raw apple pulp values. The value of color saturation delta chroma (ΔC) was calculated using formula:

(1) $$\left(\Delta C\right)=\sqrt{\left[{\left({\Delta a}^{*}\right)}^{2}+{\left({\Delta b}^{*}\right)}^{2}\right]}$$

The chroma (intensity of color) was calculated with formula:

(2) $$C^{*}=\sqrt{\left[{\left(a^{*}\right)}^{2}+{\left(b^{*}\right)}^{2}\right]}$$

The total color differences (ΔE*) between the samples were calculated according to the equation:

(3) $${\Delta E}^{*}{}_{\mathrm{ab}}=\sqrt{\left[{\left({\Delta L}^{*}\right)}^{2}+{\left({\Delta a}^{*}\right)}^{2}+{\left({\Delta b}^{*}\right)}^{2}\right]}$$

Color difference can be interpreted as follows: 0 < ΔE* < 1—differences in color are unrecognizable by a standard observer, 1 < ΔE* < 2—only an experienced observer is able to perceive the differences, 2< ΔE*< 3.5—an inexperienced observer is able to perceive the differences, 3.5 < ΔE* < 5—every observer can easily see the difference, and ΔE* > 5—an observer recognizes two different colors [48,49].

### 2.5. Physicochemical Composition

To assess the technological quality of apple products (Experiment I, II, III) that were selected based on the sensory panel and consumers, the following analyses were conducted: dry matter content, total ash content, titratable acidity, pH, total soluble solids, and vitamin C content. 

The determination of physicochemical composition was carried out in accordance with Polish standards:

- dry matter content, the samples were oven-dried at 70 °C until a constant weight was attained. DM was expressed as percentage dry weight of the initial fresh sample;
- total ash content [50];
- titratable acidity [51]. Results were expressed as mg g-1 of malic acid equivalent (MAE).
- pH was conducted following the potentiometric method and using pH-meter Knick 913 (Elektronische Messgeräte, GmbH & Co. KG, Berlin, Germany) according to [52];
- total soluble solids content was determined using the refractometric method [53].

Determination of vitamin C content was conducted by a high-performance liquid chromatography system using a Waters chromatography (Waters Corporation, Milford, MA, USA) paired with a UV-VIS detector (2487, Waters Corporation, Milford, MA, USA) at λ = 245 nm wavelength. Chromatographic separation was performed using the Symmetry RP C18 5 μm column (Waters Corporation, Milford, MA, USA) and a mobile phase flow rate kept at 0.8 mL/min. Vitamin C (total content of L-ascorbic and dehydroascorbic acid) was extracted from the samples using a metaphosphoric acid extraction reagent. To determine the total content of vitamin C, a reduction of dehydroascorbic acid to ascorbic acid was carried out in the presence of a reducing substance (DTT) and a mobile phase flow of 0.8 mL/min. Calibration curves were made and vitamin C content was calculated on this basis. All analyses were conducted in triplicate.

### 2.6. Data Analysis

The statistical analysis of the results was performed using Statistica software version 13.1 PL (StatSoft, Krakow, Poland). The mean of the results, standard deviation (SD), one-way analysis of variance (ANOVA) with Fisher’s least significant difference (LSD) post hoc test, as well as the coefficient of correlation according to Pearson were all calculated. Statistical significance was defined at *p* ≤ 0.05. In the variance analysis, the effect of SGs concentration and food additives use (citric acid or citric acid and pectin addition) on sensory quality and physicochemical composition of preserves was measured. The results of sensory profiling of processed apples were analyzed by principal components analysis (PCA), which enables the establishment and better representation of dependencies in the data set [54]. 

## 3. Results

### 3.1. Optimization of the Level of Steviol Glycosides Addition to Apple Preserves Taking into Account Sensory Properties

**Figure 2:**
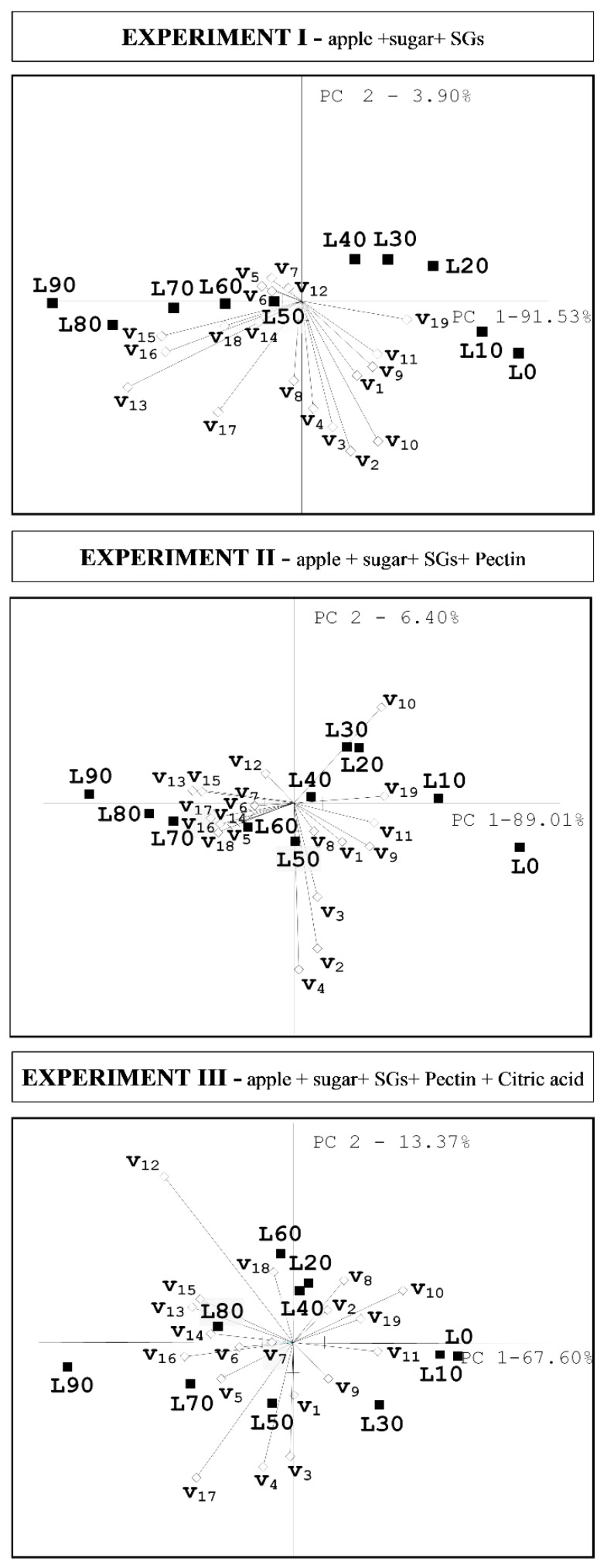
Principal components analysis (PCA) biplot of apple preserves’ sensory profiles. L0–L90— samples with 0%–90% SGs substitution; v_1_—apple o., v_2_—sweet o., v_3_—nectar o., v_4_—wine o., v_5_— metallic o., v_6_—sharp o., v_7_—other o., v_8_—color, v_9_—apple f., v_10_—sweet t., v_11_—nectar f., v_12_—sour t., v_13_—bitter t., v_14_—spicy f., v_15_—metallic f., v_16_—astringent s., v_17_—bland f., v_18_—other f., v_19_—sensory balance; o—odor, f—flavor, t—taste, s—sensation.

In general, the increase in the level of sugar substitution with the steviol glycosides resulted in a significant reduction in the intensity of apple, sweet, nectar, wine odors, and simultaneously an increase in the intensity of metallic, sharp, ‘other’ odors (ANOVA, *p* ≤ 0.05). Moreover, the intensity of sweet taste, apple and nectar flavors was significantly decreased (ANOVA, *p* ≤ 0.05), while sour and bitter tastes, astringent sensation, spicy, metallic, bland, and ‘other’ flavors increased (ANOVA, *p* ≤ 0.05). SGs addition resulted in the lower sensory balance of the preserves and was associated (*p* < 0.05) with the mentioned odor and flavor attributes (Figure 2). Despite observed tendencies, the apple preserves differed in sensory properties depending on the food additives (without, with pectin, with pectin and citric acid). Changes in intensity of some attributes, that determine the product’s characteristics and affect the harmonization of the taste and aroma, were noticed. 

The results of the descriptive quantitative analysis of the apple preserves were interpreted by using principal components analysis (Figure 2). The first two principal components explained 95.43% (in Exp. I), 95.41% (in Exp. II), and 80.97% (in Exp. III) of the total variability. The first principal component describes a negative relation between the intensity of sensory balance and negative sensory attributes for apple preserve (sharp odor, bitter taste, spicy flavor, as well as metallic or ‘other’ odors and flavors).

In Experiment I, only samples with substitution of SGs at the level of 0%–10% (L0, L10) were characterized by a high sensory balance (6.0–6.1), as well as a high note of nectar and apple flavors and apple odor, that was positively associated with intensity of sweet taste and odor. Samples with higher addition of SGs were characterized by high intensity of negative sensory attributes, like metallic and bland flavors, a bitter taste, and an astringent sensation. Sweet taste was negatively related to these attributes, which is proven by the location of those attributes on the opposite side of the plot origin. 

In Experiment II, samples with SGs added at the level of 0%–30% (L0, L10, L20, L30) also had high notes of nectar and apple flavors and odor and a high sensory balance (~6.0). Moreover, the samples with higher addition of SGs were related to negative attributes. The short vectors of negative sensory attributes reflect their low variance of the variables. The sweet taste intensity was positively associated with sensory balance. Analysis of variance revealed that the addition of pectin resulted in an intensity reduction of bland flavor as well as bitter and sweet tastes, while wine and nectar odors, sour taste, apple, and nectar flavor were enhanced (ANOVA, *p* ≤ 0.05, data not presented). The addition also contributed to the increase in the sensory balance when compared with the preserves with only sugar and SGs.

In Experiment III, the apple preserves with SGs at the level of 0%–40% (L0, L10, L20, L30, L40) had a high intensity of positive notes of odor and flavor. It was a high intensity of sweet taste and odor, nectar and apple flavors, which reflected in a higher sensory balance (>6.0). Conversely, high-level substitution resulted in an increase in the intensity of negative traits, such as astringent sensation and metallic flavor, which caused a lower sensory balance of these products (<6.0). The intensity of sweet taste was not related to intensity of sour taste as evidenced by almost right angle between positions of those vectors. However, the intensity of these tastes was negatively associated with the intensity of bland flavor, that was perceptible in the preserves with higher levels of SGs substitution. The intensity of color was also positively associated with sweet taste intensity. Statistical analysis (ANOVA, *p* ≤ 0.05) indicated that the citric acid addition in the apple preserves contributed to the increase in the intensity of apple and nectar odors and flavors, wine odor, and sour taste, as well as a decrease in intensity of ‘other’ odor and flavor, and bland flavor. Additionally, citric acid and pectin resulted in sensory balance improvement (*p* < 0.05) when compared to preserves with only sucrose and SGs addition (Experiment I) or with the pectin addition (Experiment II) (data not presented in tables). 

In conclusion, all samples of apple preserve, regardless of the additives, were characterized by high notes of sweet taste. In Experiment I, sweet taste was the most associated with L0 and L10 samples, in Experiment II with L20 and L30 samples, and in Experiment III with L20 and L40 samples. However, depending on the content of steviol glycosides and the addition of pectin or citric acid, the preserves were characterized by various other flavor notes. Their combination with high notes of sweet taste influenced the sensory properties of the product. In the preserves with high levels of steviol glycosides substitution, dominated the notes of odor and flavor attributes, that are perceived as negative, e.g., bland and metallic flavors and, astringent sensation. Such traits had a suppressing effect on the perception of sweet taste. 

### 3.2. Consumer Evaluation of Apple Preserves

Amongst consumers who tasted the apple preserves (*n* = 120), 74.8% consumed jams occasionally, and about 20% consumed preserves 2–3 times per week, 75% of those consumers were women. According to 67% of respondents, they limited sugar in their diet to stay fit (45.6%), health reasons (40.4%), or for sensory aspects (14.6%), regardless of gender and age (*p* > 0.05). 

The increase in the level of sugar substitution with the SGs was negatively correlated with an overall degree of liking of the low-sugar preserves (*r* =−0.47, *p* < 0.05). The first change in sensory-liking of the preserves (statistically significant reduction in the level) was perceivable at 20% of the SGs substitution level at all stages. The addition of pectin and pectin with citric acid increased the hedonic scores by 40% (Table 3). 

The results of the hedonic response to apple preserves (Table 3) were influenced by the frequency of jam consumption and the age of the assessors (*p* < 0.05). The highest sensory-liking scores for the preserves were found in the group of consumers that occasionally consumed jams, while the group of young consumers (18–25 years) found a higher degree of appeal in the preserves. 

Assessors that consumed a lower sugar diet (*p* ≤ 0.05) gave higher ratings to the preserves with SGs substitution (Table 3). The low-sugar preserves with high levels of sugar substitution with SGs were only rated higher by people who do not limit sugar consumption (*p* = 0.05). In general, the apple preserves with SGs substitution in all experiments were rated higher by women than men. However, men gave higher ratings to SGs substitution levels of 30%, 40%, 50%, and 60% (L30, L40, L50, L60). 

Majority of consumers indicated flavor (87.8%) and odor (47.1%) as traits that affected their evaluation the most. Color (16.9%) and consistency (23.5%) were not frequently mentioned. Less than 1% of the respondents reported ‘other’ affective factors (not presented in the tables).

### 3.3. Effect of Sugar Substitution By Sgs, on the Color of Low-Sugar Apple Preserves

In selected, sensory acceptable products (samples: L0, L10, L30, L40), color was measured instrumentally to evaluate the effect of sugar substitution with steviol glycosides on the color of the preserves (Table 4). The color parameters (L * a *, b *) of apple products were significantly affected by the percentage addition of steviol glycosides (*p* < 0.05) and the additives used (pectin, citric acid, correlations *r* = 0.80–0.96; *p* < 0.05).

Along with the increasing addition of steviol glycosides, the samples became lighter, less yellow (increase in the negative values of the Δb parameter), and less red (positive value of the Δa parameter), when compared to the control samples with only sugar. The value of the total color difference (ΔE) increased and every observer can easily see the difference between the samples. The addition of only pectin to apple products with steviol glycosides (Experiment II), changed the color to less yellow (negative Δb value), as well as less red color (negative Δa value), with the increases of SGs addition (Table 4). The most bright color (increase in ΔL) of apple products was determined in preserves with the addition of pectin and citric acid (Experiment III). These samples had the lightest color, and the value of the total color difference was almost twice higher, when compared to samples with only steviol glycosides. The addition of pectin and citric acid to products also caused a change in the color, that was greener and less red (increase in the negative value of the parameter Δa) (Table 4). The addition of citric acid stabilizes the color, the smallest changes in the ΔE parameter have been determined. The samples were also characterized by a more yellow color and the highest saturation (positive value of the Δb parameter and the highest value of the ΔC parameter) as opposed to other products that were less yellow. With a higher content of steviol glycosides, products with citric acid were more yellow in color. In summary, the color of apple preserves varied depending on the sugar and steviol glycosides content, as well as the additives used. The more sugar was added, the darker samples were. Preserves with the SGs addition were brighter than preparations with only sugar, as they caramelized during heat treatment. On the other hand, the addition of pectin and citric acid resulted in a brighter color of the preserves.

### 3.4. Effect of Sucrose Substitution with Steviol Glycosides on the Technological Quality of Apple Preserves

Products selected (L0, L10, L30, L40, in Experiment I, II, III) based on the results of sensory analysis were subjected to physicochemical tests to evaluate the effect of sugar substitution with steviol glycosides on the technological quality of the preserves (Table 5).

.

A significant effect (*p* ≤ 0.05) of the level of sucrose substitution with steviol glycosides (addition %) in apple preserves, as well as the use of additives (citric acid, pectin) for preparations on the quality (dry matter, total soluble solids, vitamin C, Ash, pH, titratable acidity, malonic acid, color L*, color a*, color b*) of the evaluated preserves was found. 

Soluble solids content in apples used for the preparations was 16.3%. Along with the increasing addition of steviol glycosides (or decreasing sucrose content) in apple preserves across experiments I, II, III, a statistical (*p* < 0.05) decrease in the total dry matter and soluble solids content was found. 

The content of vitamin C in the preserves was low and positively correlated with the content of additives (*r* = 0.41, *p* < 0.05). There was no strong correlation between vitamin C content and steviol glycosides concentration (*r* = 0.21, *p* < 0.05). 

The total ash content of apple preserves was positively correlated with the additives used (Experiment I, II, III), *r* = 0.69 (*p* < 0.05); as well as with SGs addition level, *r* = 0.64 (*p* < 0.05). The higher SGs substitution level (the lower sucrose content), the higher total ash content was revealed. 

The pH values in all samples ranged between 3.20–4.09 and was positively correlated with additives use (Experiment I, II, III), *r* = 0.69 (*p* < 0.05) and SGs substitution level, *r* = 0.64 (*p* < 0.05). 

Similar dependencies were revealed for titratable acidity and malic acid content. The titratable acidity, expressed as malic acid content, depends on the initial organic acid content. 

The content of malic acid in fresh Gloster apples was 0.42g/100 g. Along with a higher SGs’ substitution level (Experiment I, apple products with only sugar and SGs), titratable acidity and malic acid content *r* = 0.80 (*p* < 0.05) increased. 

Similar dependencies were found for apple products with pectin and citric acid addition (*p* < 0.05). In the preserves with the SGs and pectin’s addition, the acidity was reduced, while in the preserves with citric acid, it was increased when compared to the sample without additives (*p* < 0.05). 

## 4. Discussion

### 4.1. Sensory Quality of Low-Sugar Apple Preserves

The results of this study showed that high levels of sugar substitution with the steviol glycosides decreased the sensory properties of apple preserves to a level not acceptable by consumers. Higher SGs addition levels were associated with more intense sweet flavor and lighter color, a higher intensity of metallic and ‘other’ flavors and odors, as well as sharp odors, a bitter taste, astringent, spicy, and bland flavors. The perceptibility of these negative characteristics in the apple preserves reflected in their lower sensory balance. The dominant bitter taste may be caused by a decrease in sweet taste intensity at maximum SGs concentrations. This effect can be explained by intramolecular cross-modal suppression between the sweet and bitter taste components of steviol glycosides [16]. The sweet and bitter tastes, when implemented together, suppress each other. SGs have also shown a varying degree of bitterness along with intense sweet flavor [16,55]. 

Studies by Ghandehari Yazdi et al. [56] and Hemada et al. [38] were similar to our study and the results indicated that along with a higher SGs’ substitution level, the intensity of sweet and fruity flavors decreased, while metallic and licorice-like as well as off-flavors became apparent. Stevioside and RebA bind to the allosteric receptors of the sweetness receptors and inactivate them, causing the perception of sweetness to decrease. In addition, at rising SGs concentrations, a bitter licorice-like secondary or after-taste becomes increasingly apparent [16]. At higher SGs concentrations, the bitter taste may become dominant and mask the sweet taste that is dominant at a lower concentration. A bitter note was detected at the same time, which indicates that the RebA molecules bind to bitter receptors. If the ratio of the sweeteners is balanced, with 50% RebA and 50% sucrose, it can be assumed that the sweet receptors are not yet inactivated and that there are no changes in the perception of sweetness [57].

The addition of pectin resulted in an intensity reduction of bitter and sweet taste, while wine odor, nectar odor, sour taste, and nectar flavor were enhanced. The addition also contributed to the increase in the sensory balance when compared with the preserves with only sugar and SGs. The citric acid addition in the apple preserves contributed to the increase in the intensity of apple and nectar odors and flavors, wine odor and taste, as well as a decrease in intensity of ‘other’ odor and flavor, and bland flavor. Additionally, citric acid and pectin resulted in sensory balance improvement (*p* < 0.05) when compared to preserves with only sucrose and SGs addition (SGs samples) or with the pectin addition (SGs-P). The result is in agreement with Yosefi et al. [58], where the reduction of the sugar content to 50% in quince jam without any changes in its sensory properties was possible. 

The low sensory-liking of preserves with a high SGs substitution level indicates the negative effect of sugar substitution with steviol glycosides on sensory properties, which can be improved by the addition of pectin and citric acid, commonly used in the food industry. The addition of lime flavor is able to mask the side effect of *Stevia rebaudiana* which warrants future use of flavors as masking agents in stevia-sweetened products [59].

Previous studies focused on optimal levels of sugar substitution with SGs development to maintain the desired sensory quality of processed fruit products. Most of them relates to the optimization of beverage flavors. For example, sugar substitution with SGs in peach juice [60] without deterioration of sensory properties was at a lower level than in our study: 0.016 g/100 ml stevioside instead of 0.34 g/100 mL sucrose. Higher SGs substitution level was reported by Prakash et al. [14]. In fruit-flavored beverages, the acceptable flavor profile was obtained using the addition of 3% sucrose and 0.04% of SGs. On the other hand, Gałkowska et al. [61] indicated the possibility of replacing up to 60% of sucrose by SGs in lemon-flavored jellies with no significant effect on sensory quality. 

Just a few studies were aimed to design fruit jams with SGs addition; however, it is not possible to directly relate their results to this study because many other additives besides fruits, sugar, steviol glycosides, pectin, and/or citric acid were used. De Carvalho et al. [36] created a strawberry jam with cranberry juice, rebaudioside A (0.17 g/100 g of strawberries) as well as the addition of pectin, ascorbic acid, and citric acid. Samples that were sweetened either only with Reb-A or with sucralose showed similar sensory acceptance to our results. However, the jam with Reb-A had much higher hedonic scores, including flavor-liking scores, than jams with sucralose, which indicates its great technological potential. 

### 4.2. Physicochemical Properties of Low-Sugar Apple Preserves

The apple processing induces the large loss of nutrients and bioactive ingredients, mainly during pasteurization or sterilization of products. This can affect the technological quality of the product, which can be improved by the selection of appropriate processing parameters, but also by optimizing the share of ingredients in the product. 

The water content of many fruit and vegetable jams, jellies, marmalades, and preserves oscillates between 24-35% [62]. Thus, the dry matter content is an important element in the quality assessment of jams, as it correlates with the content of nutrients. The low water content indicates that the jams have a long shelf life. The authors reported different dry matter contents, depending on the raw material, sugar content, or preparation method. For example, in jam from mixed ingredients (pumpkin, strawberries, Japanese quince, cornelian cherry), the dry matter content was in the range 41.07%–45.42% [63]. Lower dry matter values were found for the 100% fruit-based preserves, without added sugar—23.48% [64] and in the dogwood preserves with 20% chokeberry addition [65].

In this study, the apple preserves with the steviol glycosides addition had a higher water content (53%–62%). It can therefore be assumed that their disadvantage may be a shorter storage time, but further storage research is required for this. It should be emphasized here that these preserves were homemade, which distinguished them from those prepared industrially. 

According to Nieć et al. [64], jams prepared using the traditional method (in the ratio of fruit to sugar 2:1) had similar dry matter content of 62.8%–68.6%. Lespirand et al. [66] also indicated a higher dry matter content in homemade kiwi jams—71.2%, while it was lower in industrially prepared jams (35.4%).

The ash content of apple flesh depends on the variety. Kiczorowska et al. [67] found that the content varies from 0.39% to 0.46% of dry matter in apple pulp. In the preserves, especially only with the addition of steviol glycosides, with an increase in the content of SGs, a decrease in the total ash content was found. Thus, steviol glycosides affect the physicochemical transformations of products. However, the addition of pectin and citric acid can stabilize composition of the product. 

Apples are not a rich source of vitamin C [68]. In the flesh of various apple varieties, the content of ascorbic acid ranges within 6.0–14.5 mg/100 g [69]. Depending on the variety, the fruits contain 8.7 to 24 mg/100 g and these values were reduced by up to five times during storage [70] or by about half during processing (by comminution, spreading, packaging, and pasteurization) [71]. This is supported by the results of other authors. In the production of strawberry jam, Maceiras et al. [72] reported a 100% loss of vitamin C when compared to a fresh strawberry pulp (55 mg/100 cm^3^ vitamin C). Other authors also observed the loss of vitamin C within the production process of blueberry jam—53%–58% [73] and orange jam—52%–62% [64]. Many authors [74,75] emphasized the destructive nature of jam production, which explains the low content of vitamin C in apple preserves in this study.

This is also confirmed by the results of other authors who reported the small concentration of vitamin C in various fruit products. Higher vitamin C content was determined in fruit gels. The peach gel had 28.6 mg of vitamin C/100 g, probably due to the addition of ascorbic acid to the product. The addition of organic acids is a very common method of preserving the natural yellow-orange color of peach preserves. Other gels with fruits (without acids added) were characterized by a small amount of vitamin C, due to losses in the production process [72]. An example would be a gel with strawberries, in which the content of vitamin C was 9.1 mg/100 mL, while in fresh strawberries, it oscillates between 50–200 mg/100 mL [76].

The pH value of all products remained at a similar level, despite significant differences in the range of 3.15–4.09 (depending on the SGs substitution level). The pH values shown in this study are very similar to those found by other authors:

- in light strawberry jams—3.69 [77];
- in pineapple jam—3.56 [78];
- in orange and cherry—3.2–3.7 [64];
- in grapefruit jams—3.38 and 3.26 [79,80];
- in apple jams—3.48 and 3.52 [39].

The titratable acidity, expressed as malic acid content, depends on the initial organic acid content. It affects the flavor/taste, color brightness, stability, consistency, and product quality [81]. In fresh Gloster apples, like those used in this study, the titratable acidity was 0.42 g of malic acid per 100 g.

Low-sugar preserves made with apples of the Gloster variety have a proximate composition (dry matter, vitamin C, total acidity, pH, ash) similar to their control sample (sweetened with sugar). Although, sugar content was significantly reduced by replacing it with steviol glycosides, they were sensory accepted by the evaluators. Hence, they may be a possible alternative to products with reduced sucrose content on the consumer market. Sensory evaluation showed that Gloster apple preserve made with SGs substitution was accepted by consumers.

Abbas [35] obtained similar results from sensory and physicochemical studies of pomegranate jam with steviol glycosides substitution. Organoleptic studies of pomegranate jams have shown that the use of natural sweetener stevia as a substitute for sucrose gives sensory acceptable products. However, increasing the level of SGs’ substitution, as in apple preparations, had a significant impact on sensory characteristics. 

Our findings showed that pH, and color (a* and b*) values, increased with the decrease of the sucrose content in the jam, and the L* (lightness) value had a reversible trend. Other components (total ash content and titratable acidity) also increased. Despite significant differences in pH and acidity, these changes were relatively small [35]. The total ash content and pH values of apple preserves in this study remain at a similar level. There were no changes in the a* color value, but the L* and b* color values decreased, hence, these preparations became darker. These results are congruent with Abbas [35] conclusions. The results of the research by this author suggested that it is possible to prepare fresh pomegranate jam with good physical, chemical, sensory, and microbiological properties as well as final quality using stevia as a substitute for sucrose in all tested substitution levels and during storage of final product. 

De Carvalho et al. [36], who studied strawberry jam with the addition of cranberry juice with steviol glycosides, reported lower L* color values than in the control samples (jams with sucrose), which means a darker product. 

Only a few studies in the literature review are focused on the fruit preserves, where sugar is substituted by steviol glycosides. This fact gives this paper some significance, as well as justifying the comparison of obtained results with products of similar composition. A small number of publications on the apple preserves exhibits the need for further research on this type of product. In summary, the obtained results of physicochemical tests mostly are congruent with available reference literature, like jams from other fruits. 

Supplementary tests are needed to determine the appropriate concentrations of pectin, citric acid, and ascorbic acid in order to develop a market product. 

#### Limitations

This was a model study. The same level of pectin was used in all variants of preserves, to eliminate the effect of different levels of that additive on quality. This caused differentiation in the consistency of prepared preserves between Experiment I, II, III. Based on this reason, we did not analyze the consistency of apple preserves.

## 5. Conclusions

These results demonstrated the possibility of substituting sugar by steviol glycosides to produce energy-reduced apple preserves, with acceptable sensory characteristics and good physicochemical properties. However, it depends on the use of food additives. Application of pectin and citric acid allows the level of sugar substitution with SGs to be increased. In the preserves, without any addition of a stabilizer nor an acidity regulator, the substitution is possible only from 0% to 10% (0 to 0.05 g/100 g). The addition of pectin to the preserves allowed the SGs substitution level to increase by 30% (0.15 g/100 g). According to the analytical assessment and consumer tests’ results, sugar substitution with the SGs in the apple preserves with pectin and citric acid addition is possible up to 40% (0.20 g/100 g), without significantly diminishing the sensory balance below an acceptable level to the consumers. In these cases, replacing sugar by steviol glycosides provided acceptable products. 

Higher levels of sugar substitution with SGs (higher than 40%) reduce sensory liking to ‘unacceptable’ for consumer levels, due to flavor and odor deterioration. This is connected with a higher intensity of metallic and sharp odors, as well as a metallic flavor, bitter taste, and astringent sensation. The addition of pectin and citric acid to apple products with the addition of steviol glycosides improves the sensory balance, liking of preserves, stabilizes the color, and chemical composition. We suggest that pectin and citric acid may mask the negative effect of SGs, thus enhancing the natural flavor attributes of the apple preserves, i.e., wine or apple flavors. Further studies, including microbiological tests, are needed. 

In summary, the use of steviol glycosides has great potential in the fruit processing, low-energy foods, and foodstuff for particular nutritional uses, especially for diabetics. However, there is need to continue this research in order to develop and bring a new product to the market. These findings may be used in the future for other fruits preserves. 

Our findings may be used by the research and development department of fruit and vegetable processing plants to design new products with no added sugars and energy-reduced ones. Sugar substitution by natural ingredient—steviol glycosides in apple preserves makes possible the development of products with low sugar content which is sought by consumers.

## Figures and Tables

**Table 1:** The composition of evaluated low-sugar (L) apple preserves in 3 stages of experiments for 100 g of apple pulp.

EXPERIMENT III					
EXPERIMENT II					Citric Acid (g)
EXPERIMENT I				Pectin (g)	
Variant	Sugar (g)	Steviol Glycosides			
		(g)	(%)*		
L0	50	0.00	0	0.3	1.00
L10	45	0.05	10	0.3	0.94
L20	40	0.10	20	0.3	0.89
L30	35	0.15	30	0.3	0.83
L40	30	0.20	40	0.3	0.77
L50	25	0.25	50	0.3	0.71
L60	20	0.30	60	0.3	0.65
L70	15	0.35	70	0.3	0.59
L80	10	0.40	80	0.3	0.53
L90	5	0.45	90	0.3	0.47

* Percentage of sugar substitution with the steviol glycosides.

**Table 2:** Definition of attributes for the descriptive sensory profiling.

Sensory Traits		Definition	Word Anchors (0–10 c.u)
Odor	apple	odor characteristic for a baked apple	none → strong
	sweet	primary, refers to the presence of sugars allowing the release of a sweet aroma, sweet-smelling odor such as cotton candy, caramel or honey	
	nectar	odor characteristic for fruit nectar, honey	
	wine metallic sharp	a slightly sour odor, reminiscent of a wine acid odor, characteristic for metal (e.g., ferrous sulfate) strong, irritating, pinching (biting) odor	
	‘other’	with free space to specify by assessors	
Color		color characteristic for apple preserves	light → dark
Taste	sweet sour bitter	taste on the tongue associated with sugars taste on the tongue associated with acids taste on the tongue associated with bitter agents	none → strong
Flavor	apple	specific flavor, characteristic for baked apple	none → strong
	nectar	flavor characteristic for fruit nectar, honey	
	spicy (woody)	flavor of exotic woods mixed with bitter spice notes such as cloves, cinnamon or nutmeg	
	metallic	chemical feeling factor, specific for metal taste in the mouth or from contact with iron or copper salts (clean copper coins), an unpleasant	
	bland	a low level of flavor, and without character	
	‘other’	with free space to specify	
	astringent sensation	the shrinking or puckering of the tongue surface caused by substances such as tannins or alum	
Sensory Balance		degree of harmonization of sensory attributes; the degree to which various flavor and odor characteristics fit together in the product	low → high

c. u.—conventional unit.

**Table 3:** The results of hedonic rating of apple preserves (*n* = 120).

SGs Substitution (%)	Experiment 1	Experiment 2	Experiment 3	
	Mean of Overall Liking Scores (Hedonic Scale 1–9)			
0	6.2 a	6.8 a		7.1 a
10	6.1 a	6.0 b		6.8 a
20	4.8 a,b	6.1b		6.2 b
30	4.4 b	6.0 b		6.2 b
40	4.7 b	5.7 c		6.0 c
50	3.7 c	5.1 c		5.9c
60	3.8 c	5.2 c		5.9 c
70	3.9 c	2.8 e		5.3 b
80	2.6 d	4.3 d		5.0 d
90	1.8 e	2.9 e		3.2 e

Different letters (a,b,c, etc.) indicate significant difference in the mean values in the column at a significance level of *p* < 0.05.

**Table 4:** Color comparison of apple preserves with various SGs’ level addition $\left(\bar{x}\pm \;\mathrm{SD}\right)$.

Color (L*a*b*)	Experiment I				Experiment II				Experiment III			
	SGs (%)				SGs (%)				SGs (%)			
	0	10	30	40	0	10	30	40	0	10	30	40
L*	21.66 ± 0.19	23.18 ± 0.87	23,51 ± 0.18	25.59 ± 0.6	21.93 ± 0.87	23.15 ± 0.63	23.99 ± 0.81	26.66 ± 0.55	17.19 ± 0.60	22.27 ± 0.18	23.94 ± 0.70	24.73 ± 2.29
ΔL	-	1.52	1.85	3.93	-	1.22	2.06	4.73	-	5.08	6.75	7.54
a*	0.48 ± 0.14	0.58 ± 0.02	0.66 ± 0.20	0.65 ± 0.05	0.17 ± 0.07	0.18 ± 0.25	-0.64 ± 0.02	-0.78 ± 0.05	0.23 ± 0.24	-0.39 ± 0.02	-0.47 ± 0.04	-0.49 ± 0.06
Δa	-	0.1	0.18	0.17	-	0.01	-0.81	-0.95	-	-0.62	-0.7	-0.72
b*	13.8 ± 0.68	12.52 ± 0.44	12.13 ± 0.90	11.48 ± 0.37	13.43 ± 0.44	12.7 ± 0.28	11.01 ± 0.55	10.04 ± 0.15	7.56 ± 0.28	13.22 ± 0.24	13.37 ± 0.39	13.55 ± 0.82
Δb	-	-1.28	-1.67	-2.32	-	-0.73	-2.42	-3.39	-	5.66	5.81	5.99
C	13.81	12.53	12.15	11.50	13.43	12.7	11.03	10.07	7.56	13.22	13.38	13.56
ΔC	-	1.28	1.68	2.33	-	0.73	2.55	3.52	-	5.69	5.85	6.03
(ΔE)	-	1.99	2.5	4.57	-	1.42	3.28	5.9	-	7.63	8.93	9.66

L*—lightness, a*—redness/greenness, b*—yellowness/blueness, C—chroma, ΔC—delta chroma, ΔE—total color differences; SGs – steviol glycosides.

**Table 5:** Chemical composition of apple preserves $\left(\bar{x}\pm \;\mathrm{SD}\right)$.

Experiment	SGs (%)	Dry Matter (%)	Total Soluble Solids (%)	Vitamin C (mg/100 g)	Total Ash (%)	pH	Titratable Acidity (°)	Malic Acid(g/100 g)
I	0	47.3 ± 0.2 A,a	46.0 ± 0.5 A,a	0.52 ± 0.02 A,a	0.160 ± 0.002 A,a	3.79 ± 0.01 A,a	4.92 ± 0.02 A,a	0.330 ± 0.000 A,a
	10	45.0 ± 0.3 A,b	44.4 ± 0.5 A,b	0.50 ± 0.01 A,b	0.162 ± 0.002 A,a	3.76 ± 0.01 A,b	5.28 ± 0.02 A,b	0.354 ± 0.001 A,b
	30	40.2 ± 0.4 A,c	41.2 ± 0.2 A,c	0.48 ± 0.02 A,b	0.168 ± 0.002 A,b	3.68 ± 0.01 A,c	5.99 ± 0.01 A,c	0.401 ± 0.001 A,c
	40	39.9 ± 0.3 A,c	40.8 ± 0.2 A,d	0.51 ± 0.01 A,b	0.182 ± 0.002 A,c	3.60 ± 0.02 A,d	5.97 ± 0.01 A,c	0.400 ± 0.001 A,c
II	0	48.5 ± 0.2 B,a	47.5 ± 0.4 B,a	0.55 ± 0.01 B,a	0.091 ± 0.001 B,a	3.68 ± 0.02 B,a	3.87 ± 0.01 B,a	0.259 ± 0.001 B,a
	10	47.3 ± 0.2 B,b	47.7 ± 0.3 B,a	0.56 ± 0.01 B,a	0.148 ± 0.029 B,b	3.66 ± 0.02 B,a	4.26 ± 0.02 B,b	0.286 ± 0.002 B,b
	30	47.7 ± 0.6 B,b	43.5 ± 0.5 B,b	0.54 ± 0.02 B,a	0.174 ± 0.001 B,c	3.61 ± 0.02 B,b	5.07 ± 0.02 B,c	0.339 ± 0.001 B,c
	40	42.1 ± 0.6 B,c	38.9 ± 0.2 B,c	0.55 ± 0.01 B,a	0.173 ± 0.003 B,c	3.60 ± 0.01 A,b	5.13 ± 0.02 B,d	0.344 ± 0.00 B,d
III	0	41.4 ± 0.2 C,a	45.2 ± 0.7 C,a	0.56 ± 0.02 B,a	0.161 ± 0.021 A,a	3.29 ± 0.02 C,a	10.69 ± 0.04 C,a	0.714 ± 0.004 C,a
	10	40.4 ± 0.1 C,b	43.9 ± 1.1 C,b	0.54 ± 0.02 B,a	0.163 ± 0.006 A,a	3.26 ± 0.01 C,a	12.12 ± 0.04 C,b	0.811 ± 0.004 C,b
	30	38.6 ± 0.3 C,c	39.0 ± 0.5 C,c	0.53 ± 0.01 B,a	0.167 ± 0.004 C,a	3.21 ± 0.01 C,b	14.98 ± 0.02 C,c	1.000 ± 0.001 C,c
	40	34.7 ± 0.3 C,d	36.0 ± 0.6 C,d	0.54 ± 0.03 B,a	0.169 ± 0.010 C,a	3.23 ± 0.00 B,c	15.10 ± 0.10 C,d	1.012 ± 0.008 C,d

Different uppercase letters (A, B, C, etc.) indicate a significant difference in the mean values in the column (between I, II, III experiment) at a significance level of *p* < 0.05. Different lowercase letters (a, b, c, etc.) indicate a significant difference in the mean values in the column (between levels 0%, 10%, 30%, 40% of SGs) at a significance level of *p* < 0.05.
